# Application and Research Prospects of CRISPR/Cas Gene Editing Technology in Lactic Acid Bacteria

**DOI:** 10.3390/microorganisms14040739

**Published:** 2026-03-26

**Authors:** Erhong Zhang, Jiao Yan, Jiahao Du, Xiao Chu, Dahua Chen

**Affiliations:** 1 Institute of Biomedical Research, Yunnan University, Kunming 650500, China; 2 Southwest United Graduate School, Kunming 650500, China; 3 Institute of International Rivers and Eco-Security, Yunnan University, Kunming 650500, China; 4 State Key Laboratory of Organ Regeneration and Reconstruction, Institute of Zoology, Chinese Academy of Sciences, Beijing 100101, China

**Keywords:** genetic engineering, gene editing technology, lactic acid bacteria, CRISPR/Cas

## Abstract

Lactic acid bacteria (LAB) are pivotal microorganisms in the food industry. Current approaches for functional gene validation and trait improvement in LAB primarily rely on traditional gene editing and homologous recombination techniques. These methods are often cumbersome, inefficient, and time-consuming, hindering the rapid and precise customization of strains. This limitation has, to some extent, constrained the rapid selection and industrial application of functional LAB strains. The engineering of LAB through gene editing technologies has significantly advanced both fundamental and applied research. Among these, CRISPR/Cas gene editing has successfully achieved precise modification of multiple genes in various LAB species. Compared to conventional methods, it offers superior editing efficiency and lower operational costs, opening new avenues for functional gene identification and genetic improvement in LAB. However, the application of exogenous CRISPR/Cas systems in LAB faces technical challenges such as high off-target rates, chromosomal abnormalities, and cytotoxicity. The development of endogenous CRISPR/Cas-based editing tools for LAB provides novel pathways for precise regulation, rational design, and flexible application. This paper first outlines the structural components and mechanistic principles of CRISPR/Cas gene editing tools. It then explores the research progress and applications of both endogenous and exogenous CRISPR/Cas systems in LAB. Finally, it provides an outlook on the future application of CRISPR/Cas gene editing technology in LAB, offering a reference for its implementation in this field. The advent of gene editing technologies has significantly propelled functional gene validation and trait improvement in lactic acid bacteria (LAB), thereby advancing both fundamental research and industrial applications. Notably, the CRISPR/Cas system has emerged as a transformative tool enabling precise genetic modification in diverse LAB species, offering marked improvements in editing efficiency and cost reduction relative to conventional approaches. CRISPR/Cas-based editing strategies in LAB are broadly classified into exogenous and endogenous systems. Exogenous systems operate independently of the host’s native immune repertoire, conferring the advantages of broad strain applicability and high editing efficiency. These systems have been successfully deployed for functional gene characterization, metabolic pathway engineering, such as augmenting antimicrobial production, and probiotic safety enhancement via virulence gene deletion. Conversely, endogenous systems leverage the intrinsic CRISPR/Cas machinery of LAB, offering superior biocompatibility and minimized off-target risks. Notable applications include precise gene knockout and integration using the native Type I-E system in *Lacticaseibacillus paracasei*. This review provides a concise overview of CRISPR/Cas system architecture and mechanisms, followed by a systematic synthesis of research progress and applications for both exogenous and endogenous systems in LAB. Finally, future directions are outlined to guide the continued development and application of CRISPR/Cas technologies in this field.

## 1. Introduction

Rapid advances in genetic engineering and bioinformatics have enabled researchers to gain deep insights into the intrinsic functional mechanisms of microbial activity. As a result, the application of microorganisms has entered the 3.0 era, in which modern biotechnology is used to generate genetically engineered microorganisms through the “Design-Construct-Evaluate-Optimize (DCEO)” or “Design-Construct-Test-Learn (DCTL)” iterative approaches, and even to synthesize bacterial genomes from scratch [1]. LAB are beneficial microorganisms widely found in traditional fermented foods. Due to their unique fermentation capabilities and health benefits, they play an indispensable and crucial role in the modern food industry and in the fields of human nutrition and health [2]. The U.S. Food and Drug Administration (FDA) has designated several strains of LAB as “generally recognized as safe” (GRAS). Currently, genetically engineered bacteria using *Escherichia coli*, *Corynebacterium glutamicum*, *Saccharomyces*, and other strains as chassis organisms have achieved rapid progress in the biosynthesis of bioactive compounds. However, their application in LAB remains in the exploratory stage.

**Figure 1:**
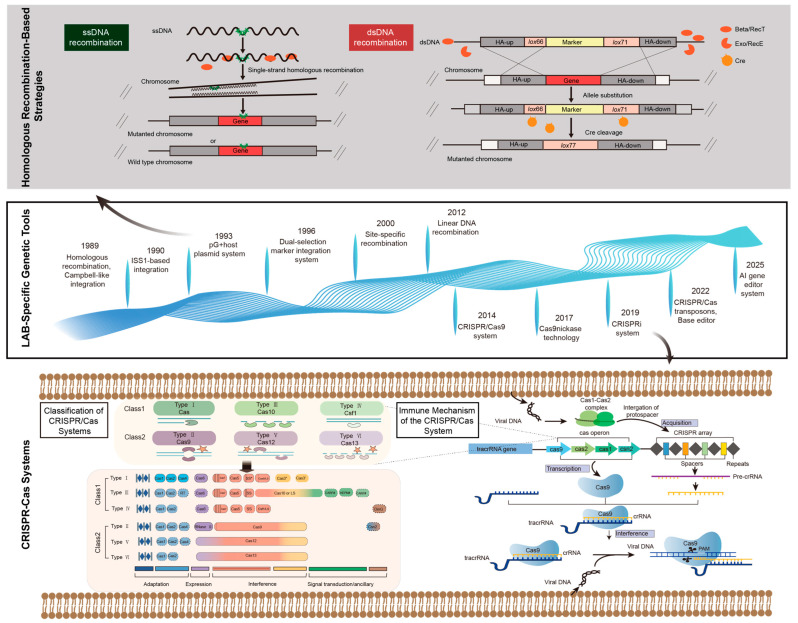
Overview of gene editing technologies in lactic acid bacteria (LAB) and the CRISPR-Cas system. The upper panel illustrates the principle of genome recombination engineering, highlighting key components: HA, homologous arm; Beta/RecT, single-stranded DNA annealing protein; Exo/RecE, 5′ → 3′ exonuclease; and Cre, cyclization recombination enzyme. The middle panel depicts the timeline of major advancements in LAB gene editing. The lower left panel presents the classification of CRISPR-Cas systems into two classes (Class 1 and Class 2) comprising six types (Types I–VI). The asterisk (*) indicates an inactivated polymerase domain essential for signal transduction, and circles represent predicted coiled-coil domains mediating protein complex formation. The lower right panel details the three stages of the classical CRISPR-Cas9 adaptive immune response: adaptation, expression, and interference.

Using molecular biology and bioinformatics techniques, researchers are gradually unraveling the mechanisms underlying the function of key genes in LAB. For example, in 1989, researchers first reported the integration of exogenous DNA into the *Lactococcus lactis* chromosome via homologous recombination, demonstrating that its Rec system (including RecBCD) could mediate homologous recombination events. This laid the foundation for subsequent gene knockout and knock-in studies in LAB. Subsequently, scientists progressively developed gene editing technologies such as Campbell-like integration systems, IS/ISS1-mediated random integration systems, pG^+^host systems, dual-selection marker integration systems, site-specific recombination, and linear DNA recombination. In 2007, Rodolphe Barrangou and colleagues experimentally demonstrated for the first time that the CRISPR/Cas system serves as an adaptive immune system against bacteriophages in *Streptococcus thermophilus*, laying the theoretical foundation for subsequent technological breakthroughs [3]. In 2012, Jinek et al. successfully engineered the CRISPR/Cas9 system from *Streptococcus pyogenes* into a programmable gene editing tool, demonstrating that CRISPR/Cas9 could cleave any specified DNA sequence in vitro [4]. In 2014, Oh and van Pijkeren et al. pioneered the use of the CRISPR/Cas9 system to achieve precise gene knockout and point mutations in *Lactobacillus reuteri* ATCC PTA 6475, elevating editing efficiency from 1% to 90% [5]. This breakthrough marked the dawn of a new era in LAB gene editing technology. Building on this foundation, researchers have developed a series of advanced gene editing tools, including Cas9 nickase (Cas9n) [6], CRISPR interference (CRISPRi) [7], CRISPR-mediated transposon systems [8], and base editors (BEs) [9]. These innovations have enabled precise genetic manipulation in LAB, significantly expanding the scope of available genetic tools and advancing their application in metabolic engineering and synthetic biology. In recent years, the rapid advancement of artificial intelligence (AI) technology has provided novel tools and approaches for LAB research. By leveraging machine learning, integrated multi-omics methods, and correlation-based strategies, scientists can not only delve deeper into the functional genes of LAB and accelerate the development of bioactive compounds but also track and simulate their dynamic responses under various environmental stresses. This enables the optimization of probiotic screening strategies and the systematic elucidation of key characteristics such as metabolic pathway regulation and gene expression changes, thereby propelling the application of LAB toward precision and systematization (Figure 1). Notably, Profluent’s recent launch of OpenCRISPR-1, the first entirely AI-generated gene editor, signals the advent of an artificial intelligence era in gene editing tool development [10].

This paper reviews the development of CRISPR/Cas gene editing technology in LAB, systematically introduces the principles and classification of CRISPR/Cas gene editing technology, summarizes the application of endogenous and exogenous CRISPR/Cas systems for targeted editing in LAB particularly in disease treatment analyzes current challenges and limitations, and offers prospects for the future development and application of CRISPR/Cas gene editing in LAB. This aims to provide a reference for LAB genetic engineering involving whole metabolic network regulation.

## 2. Overview of LAB

**Figure 2:**
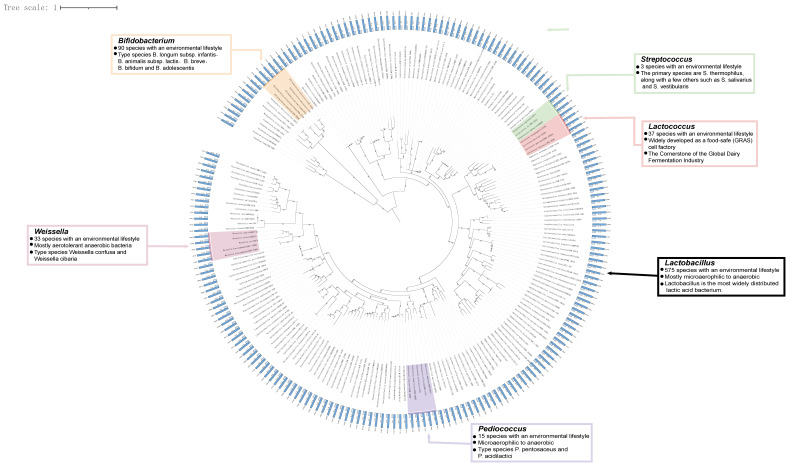
Phylogenetic relationships of representative lactic acid bacteria (LAB) strains. The phylogenetic tree was constructed based on core-genome alignments of selected LAB strains retrieved from the NCBI and IMG databases, with classification performed using GTDB-Tk (v2.4.0). The tree was inferred using the maximum likelihood method via IQ-TREE (v2.4.0) and visualized using iTOL. (A) The outermost concentric circles indicate genome size (dark blue bars) and GC content (light blue bars) for each strain. (B) Six major taxonomic groups are highlighted in distinct colors, with key characteristics summarized in the corresponding colored boxes. Representative strains from each genus were included to illustrate diversity across the LAB group.

LAB are a group of Gram-positive, non-spore-forming, facultative anaerobic microorganisms capable of producing lactic acid through fermentation. They comprise approximately 43 genera and over 373 species [11]. Broadly defined, LAB predominantly belong to the order Lactobacillales within the phylum Firmicutes, with a few species also classified under the class Actinobacteria. A phylogenetic overview of LAB genera that are commonly subjected to editing via CRISPR technology is presented in Figure 2. Members of the order Lactobacillales encompass a diverse array of LAB, including strains that are widely utilized in the fermentation of dairy products such as yogurt, vegetable products like kimchi, and meat products such as fermented sausages. Although the genus *Bifidobacterium* within the Actinobacteria class differs from LAB in metabolic pathways (heterofermentation) and genetics, it is also classified within the LAB category due to its primary production of lactic acid and similar functions. It is widely used in probiotic products [12]. Research confirms that LAB play a vital role in food fermentation and human health. In the food sector, LAB produce lactic acid, significantly lowering the pH of the environment. This effectively inhibits the growth of various pathogenic microorganisms, serving as a natural preservative and extending the shelf life of food products. Additionally, they can synthesize various bioactive substances such as vitamins, extracellular polysaccharides (EPS), and gamma-aminobutyric acid (GABA). They also hydrolyze complex macromolecules like casein and lactose into smaller peptides and amino acids that are more readily absorbed, thereby enhancing the nutritional and functional value of fermented foods [13,14,15,16,17]. In human health, the intestinal colonization ability of LAB enables them to promote the growth of beneficial microorganisms and inhibit the proliferation of harmful microorganisms by regulating the gut microbiota, enhancing adaptive immune responses, competing for nutrients (such as metallic iron ions), and secreting antibacterial compounds like bacteriocins, thereby alleviating inflammatory responses; or by regulating the secretion of host and microbial metabolites (e.g., bile salts), they serve as adjunctive therapies for conditions such as antibiotic-associated diarrhea, irritable bowel syndrome, and inflammatory bowel disease [15,18,19,20]. Simultaneously, LAB extensively participates in host development, health maintenance, behavioral regulation, and even disease onset through multiple pathways such as the gut–brain axis and gut–liver axis [14,15,16,17,18,19,20], highlighting their multifaceted role in promoting human health. The probiotic properties of certain LAB are summarized in Table 1.

## 3. Overview of the CRISPR/Cas System

### 3.1. Components of the CRISPR/Cas System

The CRISPR/Cas system is an acquired immune mechanism discovered in microorganisms during studies of microbial diversity. When phages or exogenous DNA invade a microorganism, this system captures and stores partial sequence information. Upon subsequent invasion by identical exogenous DNA, the system precisely identifies it using the previously retained sequences, subsequently cleaving and eliminating the invading DNA. CRISPR/Cas systems have been identified in over 40% of bacterial genomes and more than 90% of archaeal genomes. These systems typically comprise a set of Cas proteins and strain-specific CRISPR gene clusters [21]. CRISPR gene clusters consist of a leader sequence, highly conserved direct repeat sequences (DR), and sequence-variable spacer sequences. The leader sequence is typically a stretch rich in AT bases that plays a crucial role in the immune defense function of the CRISPR/Cas system. It contains elements associated with CRISPR transcription and harbors signals that trigger the adaptive process of the CRISPR/Cas system (acquiring new spacer sequences) [22]. Typically, CRISPR sequences across different bacteria and archaea exhibit significant variation in repeat sequences, with lengths ranging from 23 bp to 47 bp. However, for a specific bacterial strain’s CRISPR sequence, the repeat sequences are highly conserved. The length and base order of these repeats are largely consistent, with only a very small fraction exhibiting partial base differences. Most repetitive sequences contain a palindromic segment ranging from 5 bp to 7 bp in length, resulting in the transcribed repetitive sequence RNA forming a stable secondary structure. Intercalated sequences are short DNA fragments excised from invading foreign DNA, serving as a “record” of the foreign invader. They effectively prevent the re-invasion of foreign genetic elements. The length of different types of CRISPR/Cas systems varies considerably, ranging from 26 bp to 72 bp [23]. Repetitive sequences are sequentially separated by spacer sequences. Under the transcriptional activation of the leader sequence, this process facilitates the production of non-coding RNA (CRISPR RNA, crRNA). Simultaneously, the repetitive sequences are transcribed into trans-activating crRNA (tracrRNA), which together with crRNA form the sgRNA. The crRNA possesses a sequence complementary to the target DNA, enabling it to function as a guide RNA. This guides Cas proteins to cleave specific DNA sequences, achieving precise gene editing. The number of spacer sequences within CRISPR arrays and their nucleotide order reflect the temporal evolution of different strains’ responses to foreign genetic elements, with few strains sharing identical “immune processes” [24]. CRISPR spacer sequences reflect the evolutionary trajectory of bacterial strains. When a strain is invaded by an exogenous bacteriophage, new spacers are integrated chronologically into the leader region of the CRISPR sequence. By analyzing the distribution and sequence variation characteristics of spacers, the potential functions of CRISPR in LAB can be predicted, providing a foundation for utilizing CRISPR sequences to precisely study the evolution of various LAB. For example, studies combining endogenous Cas genes with repeat spacer sequences in *Lactobacillus casei* and *Lactobacillus paracasei* strains indicate that CRISPR arrays play a significant role in genomic variation within these strains [25]. Özcan et al. performed genotyping of *Streptococcus thermophilus* strains using repetitive interval sequence arrays, revealing phylogenetic relationships among strains and their ecological characteristics [26]. Rogalski et al. proposed CRISPR-based site-specific length polymorphism PCR (CLLP-PCR) [27]. Using this method, their team was able to distinguish different *Lactobacillus* strains during mixed fermentation and investigate their competitive abilities in food fermentation.

Cas proteins possess activities such as nuclease and helicase. Based on functional differences, Cas proteins can be classified into four distinct modules: adaptive regulation, expression regulation, interference suppression, and signal transduction/auxiliary modules [28]. Among different types of CRISPR/Cas systems, while the adaptor module exhibits high conservation, all other modules show significant differences. The adaptation module is responsible for integrating exogenous DNA fragments (referred to as priming sequences) into the start of the host CRISPR array. The expression modules exhibit significant differences in the processing mechanisms of precursor crRNA (pre-crRNA), featuring complex structures and diverse types. The interference module is responsible for identifying and cutting target sequences exhibiting significant structural differences. Research on signal transduction/accessory modules remains relatively limited at present. These systems primarily trigger RNA degradation by generating cyclic oligonucleotides (cOAs).

### 3.2. Classification of CRISPR/Cas Systems

Driven by the increasing abundance of genomic data and continuous advancements in bioinformatics technologies, the diversity of CRISPR/Cas systems is being progressively revealed. This diversity is not only reflected in the significant variations in Cas gene composition but also manifests in the distinct functional mechanisms that arise from these differences. In 2020, Makarova et al. classified all annotated CRISPR/Cas systems into two major categories, six types, and 33 subtypes through phylogenetic analysis, proximity analysis, and experimental data analysis, combined with the function of Cas effector proteins and the structure of CRISPR sites (Figure 1) [29]. Class 1: Ninety percent of CRISPR/Cas systems identified in bacteria and archaea (including all hyperthermophilic archaea) belong to Class 1, encompassing three types: Type I, Type III, and Type IV. These systems are characterized by effector complexes composed of multiple Cas proteins, such as the antiviral defense-associated CRISPR-associated cascade (Cascade). Class 2: Only 10% of annotated CRISPR/Cas systems belong to Class 2, encompassing three types: Type II, Type V, and Type VI. These systems are characterized by requiring only a single Cas protein to perform immune functions under crRNA guidance, such as Cas9, Cas12, or Cas13. Type I systems are widely distributed in nature. Centered around the hallmark protein Cas3, the Cascade complex, composed of three to five distinct Cas proteins, is responsible for crRNA maturation and target binding. It comprises seven subtypes: I-A, I-B, I-C, I-D, I-E, I-F, and I-G. Subtypes I-A, I-B, I-E, and I-F are widely distributed in bacteria and archaea and have been extensively studied [30]. Type III systems center on the hallmark protein Cas10, which forms a polymeric complex with other Cas proteins to cleave single-stranded DNA (ssDNA). They comprise six subtypes: III-A, III-B, III-C, III-D, III-E, and III-F [31]. All Type IV systems contain genes encoding Cas7, Cas5, and Csf1 (large subunit) proteins, encompassing three subtypes: IV-A, IV-B, and IV-C [32].

Although Type II systems are extremely rare in nature, accounting for only about 5% of bacterial systems, they have become powerful tools in genetic engineering. Compared to other systems requiring multiple Cas proteins, particularly Type II (Cas9) and Type V (Cas12a, also known as Cpf1), Type II systems are easier to reprogram due to their single-protein mechanism and can be precisely delivered to target cells. Cas9, as the hallmark protein of the Type II nuclease system, comprises two distinct nuclease domains: the HNH nuclease domain and the RuvC-like domain. These domains respectively mediate site-specific cleavage of the complementary and non-complementary strands of target DNA, generating double-stranded breaks with blunt ends. The system includes three subtypes: II-A, II-B, and II-C, along with two variants, II-C1 and II-C2. Due to its unique nucleases activity and compact enzymatic mechanism, Cas9 has rapidly emerged as a powerful tool for gene editing [33]. Cas12 is the hallmark protein of the V-type system. Unlike Cas9, it contains only a single RuvC-like domain responsible for cis-cleavage of the target strand and trans-cleavage of the non-target strand. It comprises 11 subtypes (V-A, V-B, V-C, V-D, V-E, V-F, V-G, V-H, V-I, V-K, and VU) and nine variants (V-B1, V-B2, V-F1, V-F2, V-F3, V-U1, V-U2, V-U3, and V-U4) [34]. Cas13 is the signature protein of the Type VI system, which accomplishes RNA cleavage through conserved basic residues in its two HEPN domains. This system comprises four subtypes (VI-A, VI-B, VI-C, and VI-D) and two variants (VI-B1 and VI-B2) [35]. All types function through DNA-encoded RNA-mediated nucleic acid targeting, but they differ in their mechanisms of action, molecular targets (DNA or RNA), and sequence preferences determined by the proximity of the protospacer adjacent motif (PAM). Different types of CRISPR/Cas systems contain varying numbers and types of Cas proteins, exhibiting distinct enzymatic activities at different stages of the immune response. This diversity determines the selection of systems for different application scenarios [36]. Due to its simple structure and high efficiency, CRISPR/Cas9 technology has rapidly become the preferred method for genetic manipulations such as gene knockout, knock-in, and single-base mutations in plants, animals, and microorganisms. This has opened the door to third-generation gene editing technologies and established the fundamental framework for subsequent research on the CRISPR/Cas system.

### 3.3. Mechanism of Action of the CRISPR/Cas System

Although there are numerous types of CRISPR/Cas systems, the fundamental principles of their operation are largely similar. The entire process can be broadly divided into three stages: adaptation phase, expression phase, and interference phase (Figure 1).

Phase 1: Adaptation (Spacer Acquisition). When foreign DNA invades, the Cas1 and Cas2 protein complex scans and recognizes the protospacer adjacent motif. Subsequently, the Cas1/2 protein complex cleaves a specific fragment (the protospacer) from the invading DNA and integrates it as a new spacer at the 5′ end of the CRISPR array. The CRISPR sequence integrates new exogenous DNA information and forms specific immune memory, providing the structural basis for acquired immunity. Consequently, the 5′ to 3′ arrangement of spacer regions within the CRISPR sequence also reflects the temporal sequence of exogenous genetic element invasions [23].

Phase 2: Expression (crRNA Biogenesis). Bacteria or archaea that have acquired specific immune memory transcribe CRISPR sequences into precursor crRNA (pre-crRNA) under the regulation of the leader sequence when homologous exogenous DNA fragments invade again. Pre-crRNA is processed into mature crRNA with the assistance of tracrRNA (present in systems like Type II) and various Cas proteins (e.g., Cas9, Cas4, depending on the system type). Mature crRNA, composed of spacer sequences and partial repeat sequences, can specifically recognize corresponding foreign DNA and target foreign genetic elements [37].

Phase 3: Interference (Target Cleavage). When invading foreign DNA pairs with crRNA, the bacterium’s own Cas effector protein (CEP) also forms a nucleoprotein complex with crRNA. This complex then recognizes the PAM sequence on the foreign genetic element, activating the endonuclease activity of the Cas9 nuclease. Subsequently, the two domains of the Cas9 nuclease-HNH (acting on the strand complementary to crRNA) and RuvC (acting on the non-complementary strand)—respectively cleave the foreign double-stranded DNA complementary to crRNA, causing double-strand breaks (DSBs) to achieve targeted disruption of the foreign genetic element [38]. To address DNA breaks, cells primarily employ two repair pathways: non-homologous end joining (NHEJ) and homologous recombination (HDR) repair. Gene knockout is primarily repaired via NHEJ, but this process often involves random insertion or deletion mutations, making it an imprecise repair method. While it achieves gene knockout and functional loss of the target gene, its random nature leads to uncontrollable editing outcomes, making it unsuitable for precision editing applications [39]. In contrast, gene insertion or replacement utilizes HDR for error-free repair. By introducing specific exogenous DNA templates onto the target gene, cells can repair DSBs via HDR using these templates, enabling precise insertions, replacements, and other operations on the target gene to achieve precise editing of the target gene [40]. Due to the frequent incompleteness or absence of the NHEJ pathway in LAB, gene editing in these organisms is highly dependent on HDR and exogenous repair templates.

With the advancement of research, an engineered CRISPR/Cas system has now been developed, consisting of two core components: the sgRNA (single-guide RNA) and the Cas effector protein (CEP). By adjusting the sequence of the sgRNA, the targeting location of the Cas effector protein can be flexibly altered, thereby enabling precise editing of specific target genes. Class 1 CRISPR systems, such as CEP, often exist as complexes where multiple Cas proteins collaborate to cleave target DNA, exhibiting a relatively complex interference mechanism. In contrast, the simpler structure and ease of manipulation of Class 2 Type II CRISPR/Cas9 systems have led to their widespread application in the life sciences field [41]. To achieve broader target specificity and higher editing efficiency, scientists have innovatively developed multiple CRISPR/Cas gene editing technologies and derivative tools based on the CRISPR/Cas9 gene editing technology, establishing the CRISPR/Cas system as the dominant gene editing tool today (Table 2).

## 4. CRISPR/Cas Gene Editing Systems in LAB

### 4.1. Gene Editing in LAB Using Exogenous CRISPR/Cas Systems

The editing plasmid for exogenous CRISPR/Cas-mediated genetic modification of LAB primarily comprises the following key components: an sgRNA targeting the specific gene, a CRISPR effector protein (CEP) that mediates double-strand breaks (DSBs), and a homologous repair template. This editing plasmid enables gene knock-in or knockout. Modulating the transcription levels of sgRNAs by adjusting their associated promoters can enhance editing efficiency [59]. It is worth noting that heterologous Cas9 may exhibit codon preference, leading to lower translation levels in the target bacterial strain. Therefore, codon optimization is necessary. After designing the target plasmid, select host bacteria in the logarithmic growth phase and introduce the editing plasmid into the host bacteria via electroporation to exert its function. Exogenous CRISPR/Cas systems have been employed for gene editing in certain LAB. For example, Oh et al. first demonstrated the application of CRISPR/Cas9 technology in LAB using *Lactobacillus reuteri* ATCC 6475, confirming that CRISPR/Cas9 combined with ssDNA recombination is a viable gene editing method [5]. Guo et al. combined single-stranded DNA recombination engineering with CRISPR/Cas9 reverse screening technology in *Lactococcus lactis* NZ9000, enabling efficient seamless DNA deletion or insertion within 72 h [60]. Yang et al. employed a dual-strategy approach combining double-stranded DNA (dsDNA) recombination with the CRISPR/Cas9 system to achieve targeted gene editing in *Lactobacillus plantarum* WCFS1 [61]. Zhou et al. employed CRISPR/Cas9-assisted double-stranded (dsDNA) recombination technology to perform genetic engineering modifications on *Lactobacillus plantarum* WCFS1, efficiently achieving gene knockout, insertion, and point mutation [62]. Huang et al. combined RecE/T enzymes with CRISPR/Cas9 technology to achieve single-gene knockout efficiencies of 50–100% in *Lactobacillus plantarum* WCFS1 and *Lactobacillus brevis* ATCC 367, with gene replacement efficiency reaching 35.7% [63]. Van et al. constructed a CRISPR/Cas9 integration plasmid in *Lactobacillus acidophilus* MG1363, which effectively clears plasmids, pre-phages, and integrated conjugation elements (ICEs), accelerating the construction and selection of double-exchange recombinant strains [64]. Wiull et al. constructed a CRISPR/Cas9 integration plasmid system to efficiently knock in four expression boxes ranging from approximately 800 to 1300 bp in *Lactobacillus plantarum* WCFS1 [65]. These expression boxes carry anchoring structures that mediate surface display, facilitating rapid engineering of *Lactobacillus plantarum* strains. Song et al. developed a CRISPR/Cas9 single-plasmid gene editing system for precise and efficient gene knockout in *Lactobacillus lactis* NZ9000, achieving an efficiency of up to 50% [66]. Due to significant differences in DNA repair mechanisms among various *Lactobacillus* strains, such as *Lactobacillus casei* favoring the error-prone NHEJ pathway, while *Lactobacillus plantarum* relies more heavily on the highly faithful HDR repair pathway; simultaneously, its chromosomal topology, Cas9 expression levels, and endogenous nuclease activity also influence DSB susceptibility. Furthermore, strains such as *Lactobacillus gasseri* exhibit impaired DSB repair efficiency due to the absence of the key HDR repair protein Rad51, which further exacerbates cellular toxicity. Optimizing gRNA secondary structures and introducing self-cleaving ribozymes to regulate Cas9 expression timing can also significantly enhance editing specificity and survival rates, providing a rationale for rationally designing CRISPR tools tailored to different LAB characteristics.

However, the application of CRISPR/Cas9 is often limited by the toxicity associated with Cas9-induced double-strand breaks (DSBs) in certain LAB, or by the absence of efficient DSB repair mechanisms in these strains. To address these limitations, Cas9 nickase (Cas9n) has been employed to reduce off-target effects through the generation of single-strand breaks, while catalytically dead Cas9 (dCas9) completely circumvents DNA cleavage to achieve targeted regulation without inducing genotoxic stress. Both variants, therefore, offer distinct advantages over wild-type Cas9 in preserving genomic integrity. Cas9 nickase (Cas9n) differs from the Cas9 protein through the inactivation of the HNH (H840A) or RuvC (D10A) domain. After being guided to the target site by a single-stranded guide RNA, the single domain creates a small nick in the single strand of the target gene rather than a double-strand break (DSB). Compared to Cas9-mediated double-strand breaks, the single-strand breaks (SSBs) induced by Cas9n are more readily repaired through self-homologous mechanisms [67]. For example, Song et al. established a rapid and accurate gene editing method in *Lactobacillus casei* LC2W using the CRISPR/Cas9n system, achieving an editing efficiency of 25% to 62% [6]. Kong et al. developed a CRISPR/Cas9n-assisted gene editing system in *Streptococcus thermophilus* S-3. By optimizing Cas9n and sgRNA expression using a library of natural constitutive promoters, they achieved gene knockout efficiencies ranging from 14% to 60% [68]. Li et al. performed gene deletion and insertion in *Lactobacillus paracasei* HLJ-27 using the CRISPR/Cas9n system, and the resulting recombinant strain effectively induced mucosal and humoral immune responses [69]. Chen et al. achieved *PyrR* gene knockout in *Lactobacillus casei* LC2W using the CRISPR/Cas9n system and validated the function of this gene [70]. Goh et al. utilized the CRISPR/Cas9n system to achieve targeted gene deletion, insertion of fluorescent genes, and single-base substitutions in *Lactobacillus acidophilus* NCFM, *Lactobacillus gasseri* NCK334, and *Lactobacillus paracasei* NCK2639 [71]. This demonstrated the system’s robust stability and multifunctionality, opening new avenues for developing health-promoting engineered LAB. Li et al. employed the CRISPR/Cas9n system to express the PEDV *S1* gene in the genome of *Lactobacillus paracasei* ΔAlr HLJ-27, thereby constructing the alanine-deficient engineered strain *S1*/ΔAlr HLJ-27, laying the foundation for oral vaccine development [72]. Additionally, introducing point mutations H840A in the HNH domain and D10A in the RuvC domain of the Cas9 protein yields the variant protein dCas9, which lacks endonuclease activity, rendering it incapable of double-strand cleavage. While it retains the ability to bind to target DNA sequences, it impedes RNA polymerase binding to the target gene, making it a powerful tool for gene silencing and transcriptional regulation, namely, CRISPR interference (CRISPRi) [73]. In practical applications, dCas9 can effectively suppress gene expression without requiring fusion with an inhibitor protein. CRISPRi leverages the targeting function of sgRNA, enabling the switching of target genes by altering just 20 nt of the target sequence. For example, Myrbraten et al. constructed a CRISPRi system for identifying key genes in *Lactobacillus plantarum* WCFS1. By expressing the nuclease-inactivated Cas9 (dCas9) and gene-specific single-guide RNA (sgRNA) in separate plasmids, they achieved efficient suppression of target gene expression [7]. Aleš Berlec et al. targeted and silenced the *upp* gene in *Lactobacillus lactis* NZ9000 using a CRISPRi system, resulting in a 50-fold reduction in the relative transcription level of its mRNA [74]. Xiong et al. constructed an inducible CRISPR/dCas9 gene silencing system in *Lactococcus lactis* NZ9000, enabling dynamic regulation of gene expression [75]. Tamaki et al. established a CRISPRi system in *Lactobacillus paracasei* BL23, effectively suppressing *sirA* gene expression in the recombinant strain [76]. This approach provides novel insights and tools for regulating gene expression in LAB and other microorganisms.

To overcome the bottleneck of low efficiency in inserting large DNA fragments using traditional methods, Pechenov et al. employed a CRISPR/Cas-mediated transposition mechanism to achieve efficient insertion of a 10 kbp exogenous fragment into *Lactococcus lactis* IL1403 [8]. In expanding the application of CRISPR/Cas systems for multi-target editing, researchers achieved more precise, breakage-free co-editing of multiple genes by introducing base editors, providing a significant pathway for the technology’s widespread adoption in LAB engineering. For example, Tian et al. pioneered the introduction of the CRISPR-deaminase-assisted base editor (CRISPR-DBE) system into *Lactococcus lactis* F44, achieving highly efficient, DSB-free multigene inactivation [9]. They successfully attained nearly 100% C-to-T and A-to-G editing efficiencies in *Lactococcus lactis*, providing a convenient approach for sequential gene editing. Pan et al. employed exogenous CRISPR base editors to genetically engineer *bifidobacteria*, introducing C-G to T-A mutations to form amber codon mutations [77]. This rendered multiple *bifidobacteria* strains sensitive to tetracycline, holding broad implications for developing next-generation probiotics. The development of CRISPR/Cas gene editing technologies has provided support for elucidating the functional genes and metabolic regulation of LAB, demonstrating unique application prospects in synthetic biology and functional gene research of LAB. CRISPR/Cas-mediated gene editing technologies for LAB are summarized in Table 3.

**Table 3:** CRISPR/Cas-mediated gene editing technology in LAB.

Tool	Principle	Applied Species	Application Efficiency	References
CRISPR/Cas9-Assisted Single-Stranded DNA (ssDNA) Recombination	Phage-derived single-stranded DNA-binding proteins (RecT or Beta) interact with single-stranded DNA to edit the template strand. The CRISPR system cleaves unmutated DNA, eliminating wild-type mutants	*Limosilactobacillus reuteri reuteri* ATCC6475, *Lactococcus lactis* NZ9000, *Lactiplantibacillus plantarum* WCFS1	more than 75%	[5,60,62]
CRISPR/Cas9-Assisted Double-Stranded DNA (dsDNA) Recombination	The phage-derived Exo protein binds to the exonuclease RecE, cleaving double-stranded DNA into single strands. Subsequently, the beta protein of the recombinase gene binds to the single-stranded DNA-binding protein RecT and undergoes recombination exchange with double-stranded DNA	*Lactiplantibacillus plantarum* WCFS1	53.30%	[61,62]
RecE/T-assisted CRISPR-Cas9	A versatile toolkit for recombinant helper plasmids and broad-host-range CRISPR-Cas9 editing plasmids, enabling single-gene knockout and chromosomal gene replacement	*Lactiplantibacillus plantarum* WCFS1, *Levilactobacillus brevis* ATCC367	50–100%	[63]
Integrated Plasmid CRISPR/Cas9 System	The integrated plasmid pLABTarget regulates *Streptococcus pyogenes* Cas9 expression via a constitutive promoter, enabling plug-and-play introduction of short guide RNA (sgRNA) sequences to target specific gene loci	*Lactococcus lactis* MG1363	100%	[64,65]
Single-plasmid CRISPR/Cas9 system	ITo investigate the effects of different single guide RNA (sgRNA) promoters, we optimized the efficiency of gene deletion.	*Lactococcus lactis* NZ9000	50%	[66]
CRISPR/Cas9n	Inactivated HNH (D10A) or RuvC (H840A) domains cause DNA single-strand breaks, facilitating self-homologous repair	*Lacticaseibacillus casei* LC2W, *Streptococcus thermophilus* S-3, *Lacticaseibacillus paracasei* HLJ-27, *Lactobacillus acidophilus* NCFM, *Lacticaseibacillus gasseri* NCK334, *Lacticaseibacillus paracasei* NCK2639	25–62%	[6,68,69,70,71,72]
CRISPR/dCas9	The RuvC and HNH domains are inactivated without affecting Cas9′s binding to DNA, thereby preventing RNA polymerase from binding to the promoter to achieve transcriptional repression	*Lactococcus lactis* NZ9000, *Lactiplantibacillus plantarum* WCFS1, *Lacticaseibacillus paracasei* BL23	Up to 99%	[7,74,75,76]
Transposon-mediated CRISPR/Cas systems	Insert large DNA fragments	*Lactococcus lactis* IL1403	Perform a 10 kbp insertion	[8]
CRISPR Deaminase-Assisted Base Editing (CRISPR-DBE)	Achieved C-to-T and A-to-G editing efficiency without DSB polygenic inactivation, screened natural large deletions up to 27 kb and generated a 500 bp deletion in the *tetW* gene to insert C•G → T•A amber mutations	*Lactococcus lactis* F4, *Bifidobacterium*	100%	[9,77]

### 4.2. Gene Editing Using Endogenous CRISPR/Cas Systems in LAB

The exogenous CRISPR/Cas9 system features a simple structure and a well-studied genetic background. Mature gene editing tools have been developed and successfully applied in various organisms. However, the application of exogenous CRISPR/Cas9 technology in LAB still faces certain limitations. First, constructing heterologous expression vectors is structurally complex, typically requiring approximately 9–10 kb. Endogenous elements comprise only the gRNA vector and repair template, totaling approximately 4–5 kb. Since LAB are a group of thick-cell-walled Gram-positive bacteria, larger editing plasmids generally result in lower transformation efficiency and struggle to maintain stability within these bacteria. Second, the exogenous Cas9 proteins currently in widespread use exhibit cytotoxicity toward certain LAB hosts, hindering the application of exogenous CRISPR/Cas9 technology in LAB [78]. Research has revealed that the genomes of LAB harbor abundant CRISPR/Cas system resources, with the distribution and types of these systems exhibiting significant species specificity [79]. CRISPR/Cas systems exhibit diversity among different LAB. Type II is the most prevalent CRISPR/Cas system in LAB, followed by Types I and III. To date, no Type IV, V, or VI systems have been detected [80]. Among these, the I-E and II-A subtypes are the most abundant in the *Lactobacillus* I and II systems, respectively. Type I systems, which utilize multiprotein Cascade complexes and the Cas3 nuclease, are well-suited for genome streamlining or large-fragment deletions. In contrast, the Type II-A Cas9 system is more adept at mediating precise edits involving single bases or small fragments. Therefore, the selection of an I-E or II-A subtype Cas9 system should not be based solely on technical maturity but rather involve a systematic evaluation that considers the repair preferences of the LAB host, the scale of the target editing, and the requirements of downstream applications. For instance, when constructing high-yield probiotic chassis, the I-E type is prioritized to achieve batch elimination of redundant metabolic pathways; conversely, when optimizing specific enzyme expression levels, the approach shifts to Cas9-mediated fine-tuning of promoters.

The endogenous CRISPR/Cas gene editing system in LAB offers significant advantages. It eliminates the need for expressing exogenous Cas proteins, requiring only customized gRNAs and repair templates. The resulting plasmids are of moderate size, facilitating their transfer into LAB for genetic manipulation. This simplifies the gene editing construction process and avoids potential toxicity to cells caused by exogenous Cas proteins. When editing plasmids are combined with endogenous CRISPR/Cas systems, they form highly efficient gene editing tools capable of achieving precise gene knockout, integration, replacement, single-nucleotide mutations, and the elimination of mobile genetic elements such as gene islands [81]. The process of autologous targeting using the endogenous CRISPR/Cas system in LAB more closely mimics natural mechanisms. Compared to exogenous systems, the endogenous system demonstrates superior editing efficiency. Furthermore, the endogenous CRISPR/Cas system in LAB can overcome genetic constraints, enabling the development of strains that are both safe and efficient, possess phage resistance, and can block the spread of antibiotic-resistant plasmids. This provides a novel approach for gene editing in LAB.

**Figure 3:**
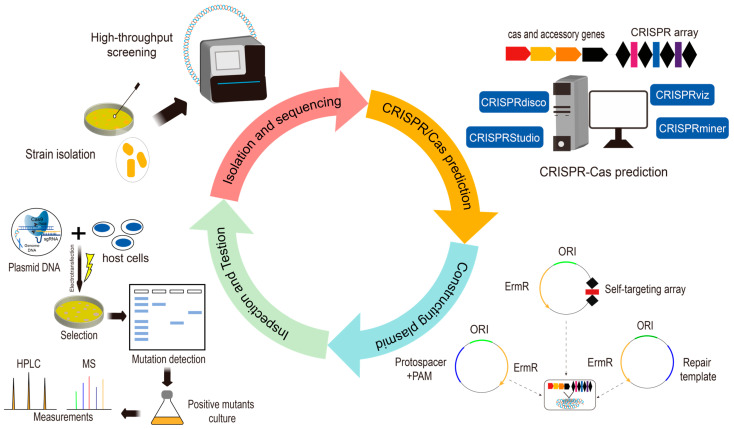
Workflow Diagram for CRISPR/Cas System Prediction and Characterization. The workflow is bifurcated into two main phases: In the first phase, a whole-genome analysis of the target strains was conducted, and bioinformatics was used to identify the types of CRISPR systems and PAM sequences present in the strains. In the second phase, plasmid interference experiments were conducted using the predicted endogenous CRISPR systems and PAM sequences. The phenotype of the host cells was used to determine whether the expected gene editing events had occurred within the cells, thereby identifying effective endogenous CRISPR systems and PAM sequences.

To perform gene editing using the endogenous CRISPR/Cas system in LAB, one must first characterize the CRISPR system within the bacterial genome and determine the PAM sequence recognized by the Cas protein. Subsequently, this information is used to construct targeting plasmids for the target gene [36], primarily involving the following steps (Figure 3): (1) Identification of CRISPR/Cas System Types: Conduct whole-genome analysis of target LAB using tools such as CRISPR/Cas Finder [82], CRISPRDetect [83], and CRISPRdb [84] to identify the CRISPR system types (Cas genes), spacer sequences, and repeat sequences present in the bacterial genome. (2) PAM Sequence Prediction and Screening: PAMs were identified using the CRISPR Target web tool [85]. Visualize base preferences at each site using WebLogo (accessed on 28 January 2026, https://weblogo.threeplusone.com/). Validate predicted PAMs through cellular experiments to assess their efficacy, construct interference plasmids containing candidate PAM libraries and transfect them into host cells. Determine whether the expected gene editing events occurred within host cells by analyzing host cell phenotypes, thereby screening for effective PAMs [86]. (3) Detecting endogenous CRISPR system activity using plasmid interference experiments: Given that the accuracy of PAM prediction is substantially affected by strain-specific variations in CRISPR adaptability, non-essential genes within the genome were selected as targets. Accordingly, interference plasmids were constructed containing an effective PAM sequence along with a novel spacer sequence designed to target the selected non-essential gene. Electroporate the plasmids into host cells. Validate the gene editing potential of the endogenous system through phenotypic analysis and PCR sequencing of transformants. (4) In vivo gene editing validation: Design targeting plasmids to verify whether gene editing can occur within the host organism. The construction of the targeting plasmid mimics the structure of the CRISPR system, incorporating a mini-CRISPR cassette and a homologous repair template into the backbone plasmid. The mini-CRISPR cassette adopts a leader-repeat-spacer-repeat structure. The targeting plasmid is electrotransfected into host cells, and positive clones are screened via antibiotic plates before undergoing further sequencing validation. Currently, only the endogenous systems of certain LAB have been characterized or developed into gene editing tools. Characterized LAB include *Streptococcus thermophilus*, *Lactobacillus gasseri*, *Bifidobacterium*, *Lactobacillus crispatus*, *Pediococcus acidilactici*, and others. For detailed information, see Table 4.

Initially, the endogenous CRISPR system in LAB was primarily applied to strain identification and precise typing. It enabled differentiation between distinct strains of the same species, elucidated phylogenetic relationships among strains, and characterized their ecological distribution patterns [25,87]. With the advancement of research, the endogenous CRISPR/Cas system is currently being utilized in LAB primarily for gene editing techniques such as point mutation, gene insertion, knockout, and transcriptional regulation. The I-E and II-A subtype CRISPR/Cas systems are widely distributed among LAB, particularly prevalent in the genus *Lactobacillus*, making them the most extensively utilized types to date. For example, the native I-E systems of *Streptococcus thermophilus*, *Lactobacillus curvatus*, and *Lactobacillus paracasei* have been engineered for efficient gene editing, including insertions, deletions, and single-base substitutions through plasmid-based recombination [81,88]. Simultaneously, the endogenous II-A systems of *Streptococcus thermophilus*, *Lactococcus lactis*, *Lactobacillus casei*, *Lactobacillus paracasei*, and *Lactobacillus rhamnosus* have also been reprogrammed for gene deletion, insertion, point mutation, and mobile genetic element clearance [89,90,91,92,93]. In addition to utilizing the optimal subtype, researchers have also begun validating the use of other subtypes. For instance, Pan et al. combined the endogenous I-G type CRISPR/Cas system of *Bifidobacterium animalis* with an exogenous CRISPR base editor for genetic engineering, achieving gene deletion and point mutations [77]. This demonstrated the feasibility of continuous editing technology and confirmed that other subtypes can also perform genetic editing in bacterial strains. Han et al. utilized the endogenous I-C type CRISPR/Cas system in *Bifidobacterium breve* FJSWX38M7 to achieve knockout, single-base substitution, and insertion of the uracil phosphoribosyltransferase (*upp*) gene [94]. This expanded the gene manipulation toolkit and provided a novel approach for functional gene analysis. The development of an endogenous CRISPR/Cas system in LAB not only underscores the importance of tailoring CRISPR gene editing strategies to specific strains but also paves the way for the development of a new generation of probiotics.

**Table 4:** Characterization and applications of the Lactobacillus CRISPR/Cas system.

Microbial Strain	CRISPR Types	Predict PAM 5′ → 3′	Application	Efficiency	References
*Lactobacillus gasseri* ATCC 33323	II-A	NTAA, TAAC	Replace the native *bshA* promoter sequence with a stronger variant to enhance its transcriptional activity, thereby improving the bile salt hydrolysis capability of the strain	Substitution: 50–90%	[95,96]
*Limosilactobacillus fermentum* ME-3	I-C. I-E, II-A, III-A				[95,97]
*Lactobacillus equicursoris* 143	I-E, II-A, III-A				[95]
*Lentilactobacillus parabuchneri DSM* 5707	II-A				[95]
*Lactobacillus jensenii* ATCC 25258	II-A	GG			[95]
*Ligilactobacillus ruminis* ATCC 25644	II-A				[95]
*Ligilactobacillus agilis* ATCC 33277	II-A				[95]
*Fructilactobacillus lindneri* DSM 20690	II-A				[95]
*Limosilactobacillus mucosae* DSM 13345	II-A				[95]
*Lactiplantibacillus pentosus* ATCC 8041	II-A	TTAAT			[95]
*Companilactobacillus farciminis* DSM 20184	II-A				[95]
*Lentilactobacillus parakefiri* DSM 10551	II-A				[95]
*Lentilactobacillus buchneri* DSM 20057	II-A	AAAA			[95,98]
*Lentilactobacillus kefiri* DSM 10550	II-A				[95]
*Ligilactobacillus animalis* ATCC 35046	II-A				[95]
*Lactobacillus kefiranofaciens* JCM 6985	II-A				[95]
*Lacticaseibacillus casei* ATCC 393	II-A, I-C, I-E	GAAAA, NGAA, TGMA, AA			[25,95]
*Lacticaseibacillus paracasei* ATCC 334	II-A, I-E	TGAAA, NGG, TTA	Validate the pre-systemic spacer adjacent motif (PAM) and custom single-guide RNA (sgRNA) expression cassette for gene editing	Knockout: 90% Insertion: 90% Single-base mutation: 50%	[88,90,99]
*Lacticaseibacillus rhamnosus* 47715	II-A	A, NGAAA	Verify the protospacer adjacent motif (PAM) and the customized single-guide RNA (sgRNA) expression cassette, thereby successfully achieving targeted genome editing	Insertion: 60–100%	[95]
*Streptococcus thermophilus*	I-E, II-A, II-C, III-A	AGG, AGAA, GGNG	Modify the nucleotide sequence of the *epsC* gene to enhance extracellular polysaccharide production, and employ self-targeting for the isolation of large-deletion variants from mixed populations, as well as for gene island excision	Kockout: 90% Insertion: 75–100% Single-base mutation: 29–80%	[91,92,93,95,100,101,102]
*Bifidobacterium*	I-C, I-E, I-U (I-G), II-A, II-C	TTC, AAC, NAAG, TAN, NGG, NNG, GCN	Knockout of uracil phosphoribosyltransferase and insertion of a novel carbohydrate hydrolase enabled the strain to degrade galactooligosaccharides, significantly enhancing its competitive advantage for carbon sources. A natural large deletion site spanning 27 kb was identified, and a 500 bp deletion was generated in the *tetW* gene, converting C•G to T•A	Kockout: 80%; Insertion: 50–100%; Mutation: 29–80%; Substitution: 30–60%	[77,94,103,104,105,106,107]
*Lactobacillus helveticus* ATCC 15009	I-E, I-C, I-B, II-A				[108]
*Latilactobacillus sakei* DSM 20017	II-A, II-C	AAA, AC			[109,110]
*Tetragenococcus halophilus* DSM 20339	I-C				[111]
*Pediococcus acidilactici* ATCC 33314	II-A	NGG	Employ the endogenous CRISPR system to mediate deletion of the *pyre* gene, insertion of the *l-ldh* gene, and introduction of a point mutation in the *mpi* gene	Deletions: 68.75%; Insertions: 100%; Point mutations: 90%	[89,112]
*Weissella* NBRC 106073	I-E, II-A, III-A, III-C				[113]
*Levilactobacillus brevis* ATCC 14869	I-E, II-A, II-C	CCN, NGG, GAA			[80,114]
*Lactobacillus crispatus* DSM 20584	I-E, I-B, II-A	AAA, T, GGN	Introduce deletions, premature stop codons, and single-base substitutions within the *p-gtf* gene to achieve functional knockout or modulation of its activity	Deletions: 100%; Insertions: 36%; Single-base substitutions: 19%	[81]
*Latilactobacillus curvatus* ATCC 25601	I-E, IIA				[109]
*Latilactobacillus fuchuensis* JCM 11249	II-A				[109]
*Latilactobacillus graminis* DSM 20719	II-A				[109]
*Lactobacillus johnsonii* JCM 8792	I-E, I-C, II-A	CCN, NGG			[115]
*Lactiplantibacillus plantarum* DSM 20174	I-E, II-A	CC, GAA, TGG, CTT, GGG, CAT, CTC, CCT, CGG			[116]
*Lactobacillus delbrueckii* ATCC 9649	I-E, I-C, II-A, III-A, III-D	CTT, CTC, CAT, CCN, CCT			[117]
*Ligilactobacillus salivarius* ATCC 11741	I-B, I-C, I-E, II-A, III-A	AAT, NNWNW, GAAAAC			[118]

Note: N: Represents any nucleotide (A/T/G/C) and can be understood as a wildcard. W: Nucleotides (A or T) representing weak hydrogen bond interactions.

## 5. Applications of CRISPR/Cas Systems in LAB

As key regulators of intestinal microecological balance, LAB play vital roles in promoting host health and have wide applications in food, pharmaceuticals, and healthcare. However, the development of new LAB strains remains relatively slow. Traditional strategies primarily rely on phenotypic screening and random mutagenesis, but these approaches generally suffer from low efficiency and poor genetic stability in functional improvement, which, to some extent, hampers the development of the LAB industry. Against this backdrop, CRISPR/Cas gene editing technology provides a powerful tool for enhancing the functionality of LAB and developing new strains. This technology not only enables precise editing to endow strains with entirely new functional characteristics but also significantly enhances their existing performance. This accelerates the development of new strains and strengthens the competitive differentiation of LAB products in the market. In short, the application of CRISPR/Cas systems in LAB for disease prevention and treatment primarily manifests in the following aspects: (1) Functional customization, precisely editing genes to enhance specific probiotic functions. (2) Gene function research, using editing to study gene roles and mechanisms. (3) Diagnosis and therapy, leveraging probiotic functions or using LAB as carriers for medical applications.

### 5.1. Functional Customization of Strains

CRISPR/Cas technology, leveraging its targeted editing capabilities, enables customized functional modification of LAB strains by precisely modifying key sites such as metabolic genes and functional genes. This provides a precise regulatory approach for LAB development. For example, Zhou et al. achieved seamless *GlcNAc* gene editing in *Lactobacillus plantarum* WCFS1 using CRISPR/Cas9, increasing GlcNAc yield to 797.3 mg/L and thereby expanding the application of *Lactobacillus plantarum* in the pharmaceutical field [62]. Wiull et al. constructed an inducible dual-plasmid CRISPR/Cas9 system to express the receptor-binding domain (RBD) of SARS-CoV-2 in *Lactobacillus plantarum* WCFS1 [65]. This expression cassette, equipped with an anchoring structure that mediates surface display, enhances the strain’s ability to colonize the gut, thereby effectively interfering with pathogen growth. Kong et al. employed the CRISPR/Cas9n system to perform key gene editing in the extracellular polysaccharide (EPS) biosynthesis pathway of *Streptococcus thermophilus* S-3 [68]. This intervention induced alterations in EPS molecular weight, viscosity, and monosaccharide composition, thereby enhancing the viscosity and texture of yogurt products. Their work also advanced research into the metabolic pathways of target products in *Streptococcus thermophilus*. Li et al. utilized the CRISPR/Cas9n system to construct a recombinant strain of *Lactobacillus paracasei* HLJ-27 expressing the porcine rotavirus capsid protein *VP4* gene [69]. This recombinant strain effectively induced both mucosal and humoral immune responses against porcine epidemic diarrhea virus (PEDV) infection. Li et al. employed the CRISPR/Cas9n system to express the PEDV *S1* gene in the genome of *Lactobacillus paracasei* ΔAlr HLJ-27, thereby constructing the alanine-deficient engineered strain *S1*/ΔAlr HLJ-27 [72]. Immunization and challenge tests were conducted to evaluate its immune response levels and protective efficacy against PEDV. Experiments demonstrated that oral inoculation of mice and piglets with the *S1*/△Alr HLJ-27 strain induced mucosal, humoral, and cellular immune responses. The strain also exhibited a degree of resistance against PEDV infection in piglets, achieving therapeutic molecular targeted delivery and conditional activation. This approach provides insights and methodologies for studying animal immune regulation. Pan et al. successfully conferred tetracycline susceptibility to *Bifidobacterium* using an endogenous I-G type CRISPR/Cas system [77]. This finding not only sheds light on the antibiotic resistance mechanisms in *Bifidobacterium* but also offers novel approaches to addressing strain resistance issues. Furthermore, it provides new strategies for developing novel antibiotics and probiotic therapies. Tian et al. utilized CRISPR/Cas9 gene editing technology to modify *Lactobacillus paracasei* NCBIO01-M2, generating an engineered strain capable of highly efficient L-lactic acid production [119]. This strain effectively regulates intestinal microecological balance by promoting lactic acid synthesis, thereby treating disorders such as intestinal dysfunction. Hasan et al. employed CRISPR/Cas9 technology combined with the λ-Red recombination system to target the d-lactic dehydrogenase (*ldh*) gene in *Lactobacillus bulgaricus*, providing a novel strategy to enhance its resistance to pathogenic bacteria and strengthen its antibacterial activity [120].

### 5.2. Gene Function Research

CRISPR/Cas technology can also precisely regulate specific genes in the metabolic pathways of LAB to explore the functions and mechanisms of action of these genes. For example, Myrbråten et al. utilized CRISPRi in *Lactobacillus plantarum* WCSF1 to investigate the expression levels of the *Acm2*, *DnaA*, and *EzrA* genes during bacterial growth, revealing that *DnaA* and *EzrA* play crucial roles in cell division and growth regulation [7]. Chen et al. constructed a *PyrR*-deficient *Lactobacillus casei* strain LC2W using CRISPR/Cas9D10A, revealing the regulatory mechanisms of this gene in pyrimidine biosynthesis and specific metabolic pathways, and confirming its crucial role in conferring antibacterial activity to *Lactobacillus casei* [70]. Xiong et al. developed an inducible CRISPR/dCas9-based dual-plasmid gene transcription suppression system in *Lactococcus lactis* NZ9000 [75]. They identified the bile salt hydrolase encoded by the *llnz*_07335 gene as a key factor for bile salt tolerance in *Lactococcus lactis*, revealing its environmental adaptation mechanism. Fang et al. functionally characterized the uracil phosphoribosyl transferase-related gene of *Lactobacillus paracasei* CGMCC4691 using the CRISPR/Cas9 system, identifying key genes that influence the strain’s competitive performance and enhancing the competitiveness of *Lactobacillus paracasei* against pathogenic bacteria [121]. Lemay et al. successfully conferred resistance to the p2 bacteriophage in *Lactococcus lactis* MG1363 by targeted knockout of specific genes using CRISPR/Cas9 technology, providing new insights into the molecular mechanisms underlying bacteriophage–host interactions [122]. Wang et al. employed CRISPR/Cas9 gene editing technology to construct single, double, and triple bile salt hydrolase (BSH) knockout strains in *Lactobacillus plantarum* AR113. This study investigated the roles of different *bsh* genes in bile salt tolerance, providing a scientific basis for screening strains with high bile salt tolerance [123].

### 5.3. Diagnosis and Treatment of Diseases

LAB demonstrate broad application prospects in preventing and treating various diseases. For instance, using CRISPR/Cas9 technology to precisely integrate fluorescent protein genes into the LAB genome enables specific strain labeling. By observing the distribution and dynamic changes in fluorescently labeled LAB within the body, their mechanisms of action during disease treatment can be elucidated, providing a basis for optimizing LAB therapeutic regimens. For example, Song et al. utilized CRISPR/Cas9 to insert an enhanced green fluorescent protein (eGFP) expression cassette into the *Lactobacillus casei* LC2W_1628 locus, achieving a chromosomal insertion efficiency of 25% to 62% [6]. They explored its potential applications in other LAB (LAB), laying the groundwork for investigating its role in the prevention and treatment of intestinal diseases. Li et al. utilized the CRISPR/Cas9 system to insert the constitutively expressed fluorescent reporter protein mCherry into the chromosomal genome of *Lactobacillus plantarum* WCFS1, achieving fluorescent labeling of the strain [65]. This enables direct visualization of its distribution and migration dynamics within the body, providing an effective approach for elucidating the in vivo transport characteristics and functional evaluation of probiotics. Goh et al. employed the CRISPR/Cas9n system to insert the mCherry fluorescent protein gene downstream of the *pgm* gene in *Lactobacillus acidophilus* NCFM [71]. They investigated the transport mechanism and probiotic functions of *Lactobacillus acidophilus* within the host, thereby pioneering a novel approach for the engineered modification of health-promoting probiotic LAB. Hidalgo-Cantabrana et al. successfully inserted a green fluorescent protein reporter gene (insert fragment: 730 bp; efficiency: 23%) into the endogenous Type I-E CRISPR/Cas system of *Lactobacillus curvatus*, investigating its role in preventing and treating intestinal diseases [81]. This provides a reference for developing biotherapeutic applications. The integration of CRISPR/Cas systems with fluorescent localization technology combines the precise gene editing capabilities of CRISPR/Cas systems with the intuitive visualization advantages of fluorescent labeling, opening new perspectives for the development and utilization of probiotics. In disease treatment, this technology can enhance therapeutic efficacy by precisely editing LAB genes. For instance, it can knock out or activate specific genes to regulate metabolic pathways, strengthen immune-modulating functions, or enable bacteria to serve as drug carriers for precise targeted delivery and controlled release. This approach improves treatment outcomes while reducing adverse reactions, thereby exerting therapeutic effects. For example, Yu et al. employed CRISPR/Cas9 gene editing technology in *Lactobacillus rhamnosus* GG to construct a multifunctional, self-propelled nanosystem [124]. This system precisely silenced the indoleamine 2,3-dioxygenase-1 (*IDO1*) gene, thereby activating the immune cell death (ICD) mechanism. This approach reversed tumor immune suppression to achieve therapeutic effects against tumors. Khosravi et al. successfully expressed a fusion gene encoding guanylate cyclase C and dendritic cell-targeting peptide in *Lactobacillus casei* LC2W using the CRISPR/Cas9 system, constructing a recombinant bacterium as a therapeutic vaccine against colorectal cancer (CRC) [125].

## 6. Prospects for CRISPR/Cas Systems in LAB Genome Editing

CRISPR/Cas gene editing technology, with its high efficiency, precision, and programmability, has emerged as a revolutionary tool in the life sciences. It not only provides critical technical support for fundamental research on LAB but also demonstrates immense potential in customized functional design of strains, development of live bacterial vaccines, and performance enhancement of industrial strains. This technology is propelling applied research on LAB toward a new phase of precision and systematization. Despite the remarkable achievements of CRISPR/Cas gene editing technology in LAB, its practical application still faces numerous challenges. First, at the technical level, off-target effects remain a key factor limiting its application. Although methods such as optimizing sgRNA design and using high-fidelity Cas protein variants have reduced off-target rates, risks persist in complex genomic contexts. Stout et al. screened for Cas9-targeted escape strains by introducing interference plasmids into *Lactobacillus gasseri* JG141, identifying the deletion of spacer sequences as one cause of Cas9-mediated escape [126]. Second, chromosomal structural abnormalities induced by gene editing, including segmental deletions and genomic breaks, along with the cytotoxicity of exogenous Cas9 proteins, cannot be overlooked. Cullot et al. demonstrated that CRISPR/Cas9 technology can cause large-scale chromosomal deletions spanning millions of bases [127]. Tamaki et al. found that sustained expression of Cas9 significantly suppressed host bacterial survival, indicating that the protein itself exhibits a certain degree of cytotoxicity toward cells [76]. Furthermore, the diversity of strain genetic backgrounds also limits the universality of editing tools. Leenay et al. demonstrated the impact of strain specificity on editing efficiency by comparing the editing efficiency of SpyCas9 across different *Lactobacillus plantarum* strains (WCFS1, WJL, NIZO2877) [128]. Finally, CRISPR/Cas gene editing technology itself has certain limitations, such as its dependence on PAM sequences, which restrict the freedom of target selection; single-stranded sgRNA-guided CRISPR activation systems often exhibit insufficient activity in LAB. Furthermore, plasmid-based expression systems demonstrate instability in the absence of antibiotic pressure, while chromosomal integration frequently faces challenges of low expression levels. Multigenetic editing technology struggles to address the synergistic regulation of complex metabolic networks. More importantly, biosafety and ethical controversies also pose significant challenges. The uncertainty surrounding the genetic information of engineered bacteria makes them difficult to assess in environmental release risk evaluations, while “designer microbes” challenge the ethical boundaries of human intervention in natural evolution. Currently, no unified legal regulatory framework has been established internationally.

To address the above challenges, future research may focus on the following areas: (1) Innovative Editing Tools: Harnessing the endogenous CRISPR/Cas systems of LAB (e.g., the Type I system in *Lactobacillus delbrueckii*), this self-targeting editing strategy eliminates the need for exogenous Cas proteins. It effectively avoids cytotoxicity and immunogenicity while simplifying construction. Simultaneously, actively screened novel Cas protein variants (such as Cas12, Cas13, etc.) with enhanced PAM specificity and higher fidelity broaden the targeting range and improve safety. Currently, base editors, promoter editors, and CRISPR activation/inhibition systems (CRISPRa/i) have achieved high-precision gene regulation without inducing double-strand DNA breaks. Coupled with biosensors, this approach enables the development of intelligent probiotics or industrial microorganisms capable of real-time environmental sensing and automatic metabolic flux adjustment, laying the foundation for achieving dynamically adaptive metabolic pathways in LAB. (2) Omics Integration: Omics technologies (such as genomics, transcriptomics, proteomics, metabolomics, and multi-omics analysis) provide unprecedented depth, breadth, and predictability for safety assessments of genetically engineered LAB. It shifts safety assessment from traditional phenotypic assays (such as acute toxicity and mutagenicity) toward a molecular mechanism-based “safety by design” paradigm. By integrating omics within the design-build-test-learn cycle, it not only enables more effective risk identification and mitigation but also facilitates the design of inherently safe engineered LAB, accelerating their transition from laboratory to practical application. (3) Integration of AI Technologies: With the rapid advancement of AI technology, its applications in precise sgRNA design, off-target effect prediction, annotation and discovery of novel CRISPR/Cas systems, and design and modification of key editing proteins have become pivotal drivers propelling progress in this field. Future research should focus on constructing more diverse and large-scale training datasets and developing more advanced AI models by integrating techniques such as transfer learning and ensemble learning. Examples include CRISPRedict [129], DeepFM-Crisp [130], CRISPR-GPT [131] and GLiDe [132], aiming to enhance model generalization capabilities and achieve species-specific sgRNA design. With the rise in deep learning tools such as AlphaFold [133], researchers can now rapidly discover enzyme components with unique functions using AI-driven novel protein clustering methods. This approach significantly enhances the efficiency of Cas protein and methyltransferase structure prediction and functional annotation. Meanwhile, with the advancement of protein language models, the modification of key proteins will become more precise and intelligent. A new era is about to begin, where language models will be used to design and generate entirely novel gene-editing key protein components from scratch. Additionally, with the aid of automated facilities, the entire gene editing process will be capable of high-throughput parallel operation, enabling automation across all stages from sgRNA design and experimental construction to result analysis. This approach not only significantly shortens the experimental cycle but also enables the continuous output of standardized, large-scale, high-quality data, offering a promising solution to the challenges of data sparsity and heterogeneity. In the discovery and optimization of key proteins, automated protein expression, purification, and functional testing will significantly accelerate the modification process of important enzyme components, promising to provide a continuous stream of high-quality data support for training machine learning models. Through the deep integration of automated facilities and AI technology, we can not only enhance the efficiency of gene editing but also significantly shorten the time required for technological iteration. This will enable gene editing to enter a new era of intelligent and precision-driven development at an earlier stage. (4) Biological Containment Strategies: Strategies such as nutrient-deficient strains or CRISPR-based “kill switches” are crucial for preventing modified LAB strains from persisting unintentionally in the environment or human microbiome. Current regulatory frameworks are undergoing a paradigm shift from “technological determinism” to “risk-based regulation.” For “seamless editing” strains involving only endogenous gene modification with no residual exogenous DNA, regulatory intensity will gradually ease. This development paves the way for broader applications of CRISPR-edited LAB as probiotics or fermentation agents.

The application of these platforms in microbial cell factories enables the optimization of complex metabolic networks, the construction of combinatorial libraries, and the dynamic regulation of synthetic metabolic pathways. In the future, as synthetic biology, artificial intelligence, and multi-omics further converge, the safety design and assessment of genetically engineered LAB will become more precise and efficient. Despite ongoing challenges in terms of universality, security, and regulatory compliance, these issues will be progressively addressed through continuous tool innovation, technological integration, and the refinement of ethical regulations. In summary, the increasingly sophisticated gene editing technology system for LAB characterized by its diversity, intelligence, and precision, will ultimately enable us to design next-generation strains with enhanced fermentation performance, superior colonization capabilities, and customized therapeutic functions. This advancement will serve as a core driver for the green transformation of the food industry, precision medicine for human health, and sustainable biomanufacturing.

## Figures and Tables

**Table 1:** Probiotic Properties of Selected Lactic Acid Bacteria.

Microbial Strain	Product	Mechanism/Metabolite	Efficacy	**Common CRISPR Target Genes**	**References**
*Lactobacillus*, *Lactococcus*, *Leuconostoc, Pediococcus*, *Bifidobacterium*	organic acids	Produce lactic acid, diacetyl and acetic acid	Lower pH inhibits spoilage bacteria, generates flavor compounds, promotes gel formation, and improves texture	*lacz*, *pyre*, *recA*, *upp*, *bsh*, *thyA*, *eps*, et al.	[13]
*Lactococcus lactis* MG1363, *Lactobacillus helveticus* R0052, *Lactobacillus delbrueckii* subsp. *Bulgaricus* ATCC 11842, *Streptococcus thermophilus* MG 18311, *Lactobacillus acidophilus* NCFM, *Bifidobacterium bifidum* BB-12, *Lactobacillus rhamnosus* GG	Protease, β-galactosidase	Break down macromolecular casein	Enhance protein bioavailability, reduce potential milk protein allergenicity, degrade lactose and improve lactose intolerance	*recA*, *galk*, *hemN*, *noxD*, *upp*, *d-ldh*, *eps*, *lacz*, *sod*, *IDO1*	[14]
*Streptococcus thermophilus* zlw TM11, *Lactobacillus delbrueckii* subsp. *Bulgaricus* LB-12, *Leuconostoc mesenteroides* NRRL B-512F	Dextran, fructan, heteropolysaccharide	Thicken and stabilize food texture, promote biofilm formation in bacteria	Enhance adhesion and colonization capabilities, strengthen barrier function, anti-inflammatory, immunomodulatory, antioxidant, potential cholesterol-lowering and antitumor activities	*rafE*, *lacS*, *ItaS*, *pgm*, *d-ldh*	[15]
*Lactobacillus plantarum* WCFS1, *Lactobacillus reuteri* CRL1098, *Streptococcus thermophilus* YO-MIX, *Lactococcus lactis* MG1363	Folic acid, Vitamin B12, Riboflavin, Vitamin K2	Energy production and erythropoiesis	Maintain homeostasis through synergistic interactions and participate in metabolic processes	*nagB*, *glmS1*, *rpoB*, *DnaA*, *EzrA*, *bsh*, *rafE*, *lacS*, *noxD*, *upp*	[16]
*Lactobacillus plantarum* EF1, *Lactobacillus plantarum* K154, *Lactiplantibacillus plantarum* NDC75017, *Lactobacillus brevis* GABA100, *Lactobacillus brevis* NPS-QW-145, *Levilactobacillus brevis* OPY-1, *Lactobacillus helveticus* CD6	Gamma-aminobutyric acid (GABA)	Act as a “natural tranquilizer” for the central nervous system by inhibiting neural excitation	Produce multiple physiological benefits, including anti-anxiety effects, sleep promotion, blood pressure stabilization, and neuroprotection	*nagB*, *glmS1*, *rpoB*, *DnaA*, *EzrA*, *bsh*, *ldh*	[17]
*Lactococcus lactis* MG1363, *Enterococcus faecium* AS-48, *Lactiplantibacillus plantarum* UL35, *Lactobacillus sakei* Lb706, *Pediococcus pentosaceus* ST44, *Lactobacillus reuteri* RC-14	Lacticin, Enterocin AS-48, Plantaricin, Sakacin A, Pediocin PA-1, Lactobacillin	Effectively inhibits Gram-positive bacteria, including *Listeria, Staphylococcus*, *Bacillus* (and its spores), certain Gram-negative bacteria (e.g., *Salmonella*, *Escherichia coli*) and even some fungi	Strongly chelate iron ions in the environment, forming complexes that are subsequently absorbed and utilized by the bacteria themselves	*recA*, *galk*, *hemN*, *noxD*, *upp*, *d-ldh*, *eps*, *lacz*, *sod*, *IDO1*	[18]
*Lactobacillus rhamnosus* GG, *Lactobacillus reuteri* DSM 17938, *Lactobacillus plantarum* 299v, *Lactobacillus acidophilus* NCFM, *Lactobacillus helveticus* R0052, *Bifidobacterium longum* R0175, *Bifidobacterium animalis* subsp. *Lactis* BB-12, *Lactobacillus casei* ATCC 334	Secretes mucin, regulates the function of immune cells (such as dendritic cells and T cells), and balances pro-inflammatory and anti-inflammatory factors.	Adhere to the intestinal tract and secrete antimicrobial substances	Beyond its colonization advantage, it also significantly reduces the burden of pathogenic bacteria like *Clostridium difficile* and degrades potential toxins	*IDO1*, *ldh*, *lacS*, *bsh*, *rpoB*, *URPT*, *pyrR*	[19]
*Lactococcus lactis* MG1363, *Streptococcus thermophilus Danisco*, *Lactobacillus delbrueckii* subsp. *Bulgaricus* ATCC 11842, *Lactobacillus reuteri* ATCC PTA 6475, *Lactobacillus rhamnosus* GG, *Bifidobacterium breve* M-16V, *Bifidobacterium longum* subsp. *Infantis* 35624, *Lactobacillus helveticus* R0052, *Bifidobacterium longum* R0175, *Lactobacillus plantarum* WCFS1	Cytokines, antigens, antimicrobial peptides, synthetic B vitamins, antioxidants (glutathione), and other bioactive substances	Regulate stress hormone levels, such as cortisol, and produce neuroactive substances	Reduce systemic inflammation and influence host fat absorption, glucose homeostasis and energy metabolism	*recA*, *galk*, *hemN*, *noxD*, *upp*, *d-ldh*, *eps*, *lacz*, *sod*, *IDO1*	[20]

**Table 2:** Development and optimization of CRISPR/Cas gene editing technology.

Name	Type	Dimensions (bp)	Characteristics	References
SpCas9	Cas9	1368	The first Cas9 homolog to achieve targeted mutagenesis in human cells	[41]
SaCas9	Cas9	1053	Highly efficient, specific and well-tolerated	[42]
Cpf1	Cas12a	1353	Cpf1 requires only a 42 nt tracrRNA with cleavage resulting in a distal 5′ overhang in the primary spacer region, enhancing the efficiency of NHEJ-based gene insertion. It is a single RNA-guided nuclease that requires only crRNA and contains a single RuvC domain	[43]
Cas13a (C2c2)	Cas13	—	C2c2 functions solely as an RNA-guided RNA-targeting CRISPR effector	[44]
CjpCas9	Cas9	984	CjpCas9 consists of 984 amino acid residues (smaller than SpCas9 or SaCas9)	[45]
xCas9	Cas9	1368	Enhanced PAM compatibility, significantly higher DNA specificity than SpCas9 and reduced off-target activity	[46]
SpCas9-NG	Cas9	1368	SpCas9-NG exhibits lower cleavage activity at NGG sites compared to SpCas9, enabling recognition of NG PAMs	[47]
LbCpf1 and FnCpf1	Cas12	1300	Comprising a single RuvC endonuclease domain, in plant cells, FnCpf1 edits a TTV PAM site, optimizing the Cpf1 TTTV PAM site	[48]
AaCas12b	Cas12	1129	Small size, broad genomic targeting range, low off-target activity, capable of cleaving target DNA in vitro between 4 °C and 100 °C	[49]
CasX (Cas12e)	Cas12	986	Low molecular weight, high editing efficiency, low non-specific cleavage activity	[50]
BhCas12b	Cas12	1108	The optimized BhCas12b v4 can serve as an effective programmable nuclease in various genome editing settings	[51]
SpG and SpRY	Cas9	140	SpRY nuclease and base editor variants can target nearly all PAMs, making them the most PAM-compatible Cas9 mutants available today	[52]
CasΦ (Cas12j)	Cas12	700–800	CasΦ is compact yet fully functional, combining multiple capabilities into a single protein to enable easier vector-mediated delivery and broader recognition of gene sequences	[53]
MAD7 (ErCas12a)	Cas12	1263	MAD7 exhibits a low off-target probability, possesses distinct PAM recognition sites and does not require transactivation of CRISPR RNA (tracrRNA)	[54]
AtCas9	Cas9	—	Break through PAM limitations to achieve near-PAM-free cutting	[55]
Casπ (Cas12I)	Cas12	850–867	By recognizing CCN PAMs to cleave substrate DNA, effective gene editing can be achieved in mammalian cells	[56]
Cas8-HNH	Cas8	—	Highly specific, utilizing gRNAs up to 32 base pairs in length to minimize off-target effects	[57]
Cas5-HNH	Cas5	—	Highly specific, utilizing gRNAs up to 32 base pairs in length to minimize off-target effects	[57]
AsCas12f1	Cas12	422	Low-toxicity, high-efficiency Streptomyces gene editing tool	[58]

## Data Availability

No new data were created or analyzed in this study. Data sharing is not applicable to this article.
